# Impact of Anesthesia on Brain Functional Networks in Moyamoya Disease and Spinal Lesions

**DOI:** 10.1111/cns.70358

**Published:** 2025-04-21

**Authors:** Xuanling Chen, Xuewei Qin, Zhengqian Li, Shengpei Wang, Zhenhu Liang, Hua Zhang, Lan Yao, Xiaoli Li, Ran Duan, Rong Wang, Xiangyang Guo

**Affiliations:** ^1^ Department of Anesthesiology Peking University Third Hospital Beijing China; ^2^ Department of Anesthesiology Peking University International Hospital Beijing China; ^3^ Laboratory of Brain Atlas and Brain‐Inspired Intelligence Institute of Automation, Chinese Academy of Sciences Beijing China; ^4^ Institute of Electrical Engineering Yanshan University Qinhuangdao China; ^5^ Clinical Epidemiology Research Center Peking University Third Hospital Beijing China; ^6^ The State Key Laboratory of Cognitive Neuroscience and Learning & IDG/McGovern Institute for Brain Research Beijing Normal University Beijing China; ^7^ Department of Neurosurgery Peking University International Hospital Beijing China; ^8^ Department of Neurosurgery Tiantan Hospital, Capital Medical University Beijing China

**Keywords:** brain network, intraspinal space‐occupying lesions, ischemic Moyamoya disease, propofol, resting‐state functional magnetic resonance

## Abstract

**Aims:**

To analyze the effects of intravenous propofol combined with remifentanil on whole‐brain functional networks in patients with ischemic moyamoya disease (IMMD) and intraspinal space‐occupying lesions (SOLs) using resting‐state functional magnetic resonance imaging (rs‐fMRI).

**Methods:**

Ten patients with IMMD and 10 sex‐ and age‐matched patients with lumbar SOL (normal cerebrovascular findings on preoperative MRI) were recruited. General anesthesia was administered using propofol and remifentanil. rs‐fMRI imaging was performed in both awake and anesthetized states. Whole‐brain functional network in different states was constructed based on graph theory tools.

**Results:**

In awake patients with IMMD, significant reductions in nodal strength (NS) were observed in the default mode network (DMN), sensorimotor network, and frontoparietal control network (FPN), compared to patients with SOL. Nodal efficiency (NE) showed further significant network declines. Under anesthesia, patients with IMMD: (1) exhibited disease‐specific decreases in NS and NE across several networks, potentially reflecting underlying cerebral pathology. (2) Propofol's effects also contributed to significant NS and NE reductions in several brain regions. Changes before and after anesthesia in patients with IMMD were significantly decreased in specific regions (discussed in detail) per analysis of NS versus NE. DMN connectivity correlated moderately with Montreal Cognitive Assessment scores.

**Conclusions:**

Reduced whole‐brain functional connectivity in patients with IMMD before anesthesia was similar to the alterations caused by systemic intravenous drugs administered after anesthesia.

**Trial Registration:**

ChiCTR2300075268

## Graphical abstract

Ischemic MMD alters brain network connectivity, impacting cognition and executive function. Patients with ischemic MMD under propofol combined with remifentanil anesthesia showed decreases in the DMN, SEN, FPN, and SAN. Anesthetic protocols should consider MMD‐related brain changes to minimize cognitive impact.

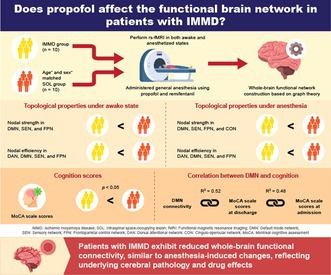

## 1 Introduction

Ischemic moyamoya disease (MMD) is a cerebrovascular disease of unknown etiology characterized by chronic progressive stenosis or occlusion of major arteries, including the middle cerebral, bilateral internal carotid, and anterior cerebral arteries [1, 2, 3]. Patients with IMMD develop early‐onset vascular cognitive changes, with a subset developing severe vascular cognitive impairment [2, 4, 5]. The cognitive impairment prevalence ranges from 23% to 71.4% [6, 7, 8, 9, 10, 11]. Vascular cognitive impairment mainly manifests in impaired intelligence, memory, and executive function [12, 13, 14, 15]. The incidence of vascular cognitive impairment in IMMD can reach 67%, with the most affected areas being information processing speed, executive ability, transient memory, visuospatial ability, and emotional disturbance. These impairments severely impact patients' quality of life [16] and corresponding brain networks.

Cognitive function is typically assessed by evaluating memory, language skills, executive function, application skills, and spatial visualization. Clinically, the Mini‐Mental State Examination (MMSE) is an effective tool for distinguishing normal cognition from dementia, although it is less effective in detecting mild cognitive impairment and assessing executive function [17]. In contrast, the Montreal Cognitive Assessment (MoCA) Scale, introduced at the European Stroke Conference in 2009, has shown greater sensitivity than the MMSE in evaluating visuospatial and executive function, verbal ability, and memory. Kasai et al. [18] also found MoCA more suitable for assessing mild cognitive impairment and dementia in patients.

Progressive stenosis of the internal carotid artery in patients with MMD decreases cerebral blood supply, and persistent chronic ischemia leads to decreased axonal density in the cerebral white matter (WM) and dendritic density in the cerebral cortex. This reduces the complexity of the cerebral neural network, decreases the connections between cerebral neurons, and damages the structural connectivity of the brain network, ultimately leading to cognitive impairment and damage to the corresponding cerebral networks and brain regions [6]. The default mode network (DMN) is an important module involved in higher cognition, especially in internally oriented, self‐relevant cognition [7], as well as in attentional and emotional control [8]. The frontoparietal network (FPN) contains two important resting‐state networks: the DMN and the executive control network (ECN). The DMN consists mainly of the posterior cingulate gyrus, medial prefrontal cortex, and bilateral parietal cortex. The posterior cingulate gyrus is strongly structurally connected to the frontal, parietal, and temporal lobes, as well as the subcortex, and is a vital node for consciousness formation [9], whereas the ECN is related to task execution [10]. Using graph‐theoretic analysis methods, Gesierich et al. [11] demonstrated that reduced functional integration of the DMN affected the cognitive domain. Cognitive impairment has been associated with changes in the right anterior/posterior cingulate gyrus and left cuneate lobe, and the effects of the disease on cognition were mediated by reduced efficiency of the DMN [12, 13], particularly in the bilateral cuneate lobe (a region commonly associated with the DMN), where processing speed and executive function correlate most strongly.

Superficial temporal artery‐middle cerebral artery direct bypass (STA‐MCA) surgery is a widely used treatment for IMMD [14, 15, 16], and requires general anesthesia. Propofol is a potent intravenous anesthetic with rapid onset and short duration of action, mainly used for induction and maintenance of general anesthesia, and is the most widely used sedative anesthetic in clinical practice [19]. Eng et al. [20] found that propofol can regulate local brain metabolism and blood flow, influencing neural activity and potentially altering consciousness by affecting regions such as the higher cortical networks and the thalamus. Oshima et al. [21]. found that propofol could proportionally reduce cerebral blood flow and oxygen metabolism while maintaining the arteriovenous oxygen partial pressure difference, ensuring normal cerebral circulation and metabolism. Both propofol and IMMD affect brain networks to different degrees [22, 23]. Fiset et al. [24] found that propofol anesthesia decreased regional cerebral blood flow in areas including the medial thalamus, cuneus, precuneus, posterior cingulate cortex, and orbitofrontal cortex. This effect was concentration‐dependent, mainly affecting brain regions related to conscious control, processing joint functions (especially visual information), and autonomic nervous system control. Schrouff et al. [25] found that propofol anesthesia diminished brain network integration, particularly within the frontal and parietal networks, affecting the level of consciousness. Although cortical sensory responsiveness is not unaffected, propofol had a modest effect on specific thalamocortical networks but showed significant inhibition of non‐specific networks [26]. Patients with MMD have significantly less functional brain connectivity than healthy controls, and impaired cerebral blood flow and brain network connectivity may be important causes of cognitive decline [27]. Despite its potential, research on propofol's effects in patients with IMMD is limited, making this an under‐explored area of current research.

The microstructural brain damage in IMMD, particularly the reduction in WM axonal density and cortical dendritic density, as well as the simplification of network complexity, is closely related to their neurocognitive dysfunction. Resting‐state functional magnetic resonance imaging (rs‐fMRI) is a valuable tool for assessing brain function and brain networks, measuring spontaneous brain activity through low‐frequency blood oxygen level‐dependent (BOLD) signals, and revealing activity correlations between brain regions induced by neuroanatomy, fluid intelligence, attention, and task execution [27, 28]. Memory‐related brain regions (such as the temporal pole, medial orbitofrontal lobe, inferior frontal gyrus, and anterior cingulate cortex) and subcortical structures consisting of the hippocampus, parahippocampal gyrus, and amygdala undergo significant decreases in fractional amplitude of low‐frequency fluctuations (fALFF) during a mildly sedated state induced by propofol. When consciousness is lost, the significant decrease in fALFF extends to the anteromedial and dorsolateral prefrontal lobes, insula, and other brain regions associated with higher cognitive control. These results indicate that the effect of propofol on BOLD signal intensity is brain region‐specific. Guldenmund et al. [29] used propofol to control the conscious state of subjects and found that with the gradual loss of consciousness, the functional brain networks of the subjects were significantly decreased by rs‐fMRI. With gradual loss of consciousness, there is a significant decrease in the connectivity between brain functional networks, mainly including the DMN, ECN, salience network (SAN), auditory network, motor network, and visual network.

Vascular cognitive impairment in patients with MMD is often due to inadequate cerebral perfusion and hypoxia [22, 29, 30, 31] and has been well documented using perfusion‐weighted MRI [22]. Abnormalities in network connectivity of the ECN, DMN, and SAN have been found in adults with MMD with vascular cognitive impairment [32, 33, 34]. The brain regions with reduced connectivity are mainly centered in the precentral gyrus, insula, precuneus, cingulate cortex, and middle frontal gyrus, which are also affected by the sedative effects of propofol. In contrast, the main brain region affected by the analgesic drug remifentanil is the right insula cortex [35], a region not central to this study. In this study, we investigated the effects of propofol on brain function and networks in patients with MMD using fMRI graph theory analysis.

## 2 Materials and Methods

### 2.1 General Patient Information

This prospective cohort study included patients with ischemic MMD (IMMD group) and space‐occupying lesions (SOLs; control group) admitted to the Department of Neurosurgery at Peking University International Hospital for STA‐MCA and space‐occupying resection of the spinal canal between May and December 2023. The study received approval from the Bio‐medical Ethics Committee of Peking University International Hospital (2023‐KY‐0016‐01). All procedures adhered to the ethical standards of the institutional and national research committee, as well as the 1964 Helsinki Declaration and its later amendments or comparable ethical standards. Informed consent was obtained from all participants.

### 2.2 Inclusion Criteria

IMMD group: (1) age 18–65 years; (2) American Society of Anesthesiologists grade I–II; (3) patients and their family members understood the study and signed the informed consent before the operation; and (4) diagnosis of IMMD. Control group: The four inclusion criteria for the IMMD group and (5) diagnosis of intraspinal SOLs.

### 2.3 Exclusion Criteria

(1) severe cardiopulmonary, hepatic, and renal impairment; (2) combined history of neurological diseases, such as epilepsy and stroke; (3) history of craniocerebral trauma or surgery; (4) recent sustained use of sedative drugs; (5) long‐term history of heavy smoking or drinking; (6) contraindications to MRI; (7) airway difficulty; (8) patient's refusal to participate in the study; (9) damage or loss of MRI data.

Patients diagnosed with intraspinal SOLs in the same period were included as a control group and underwent preoperative cranial magnetic brain examination to confirm normal cerebrovascular structure and function.

### 2.4 Anesthesia Monitoring

Upon admission to the operating room, patients underwent electrocardiogram, non‐invasive blood pressure, and oxygen saturation monitoring with an MRI‐compatible vital signs monitor (GE Healthcare Biosciences Corp., Piscataway, NJ, USA). Invasive arterial pressure monitoring was via radial arterial puncture catheterization after a negative Allen test. The first rs‐fMRI scan was performed after the completion of the monitoring.

### 2.5 Anesthesia Protocol

After completion of the rs‐fMRI scan, anesthesia was induced and maintained in both groups using the Target‐Controlled Infusion model (Braun Perfusor Space, B. Braun Melsungen AG, Hesse, Germany). Anesthesia was induced with the target plasma concentrations of propofol (4–6 μg/mL; based on the Marsh pharmacokinetic model) [36] and remifentanil (3 ng/mL; based on the Minto pharmacokinetic model) [28], and rocuronium bromide (1 mg/kg) intravenously if the Bispectral Index (BIS) dropped below 60. After induction of anesthesia and tracheal intubation, patients were mechanically ventilated with an AESTIVA MRI‐compatible anesthesia machine (GE Healthcare Biosciences Corp., Piscataway, NJ, USA), and end‐tidal carbon dioxide was monitored. Anesthesia was maintained with propofol (4–6 μg/mL) and remifentanil (4–8 ng/mL), and BIS levels were maintained between 40 and 60. After anesthesia, the BIS equipment was removed and a second rs‐fMRI scan was conducted 10 min later, once circulation and BIS stabilized.

### 2.6 Rs‐fMRI Data Acquisition

All images were acquired on a 3 T Siemens Trio MRI scanner using a 12‐channel whole‐brain coil (Siemens Medical, Erlangen, Germany). High‐resolution 3D T_1_‐weighted imaging was acquired using a magnetization‐prepared rapid acquisition gradient‐echo sequence with the following parameters: repetition time (TR) = 2200 ms, echo time (TE) = 2.55 ms, inversion time = 900 ms, flip angle = 8°, reconstructed image matrix = 256 × 256, field of view (FOV) = 200 × 200 mm^2^, 192 axial slices, slice thickness = 1 mm, and voxel size = 1 × 1 × 1 mm^3^. rs‐fMRI was collected during a 246‐volume run using an echo‐planar imaging sequence with the following parameters: TR = 2220 ms, TE = 30 ms, flip angle = 90°, reconstructed matrix = 64 × 64, FOV = 220 × 220 mm^2^, 32 axial slices, slice thickness = 3 mm with gap = 1 mm, and voxel size = 3 × 3 × 3 mm^3^.

### 2.7 Pre‐Processing

Pre‐processing was performed using statistical parametric mapping [29] and DPABI software [37] following the standardized principle and quality control procedures. The first 10 volumes in the time series were discarded to avoid the non‐equilibrium effects of signals. The functional images were slice‐timing corrected, which was performed by interpolating the voxel time using slice interpolation. Then, functional images were spatially realigned and co‐registered to their corresponding anatomical images, spatially normalized to the Montreal Neurological Institute space, and further spatially smoothed using a Gaussian kernel with 6 mm full‐width at half maximum [38]. Finally, band‐pass temporal filtering with a cutoff of 0.01–0.08 Hz was respectively obtained to reduce high‐frequency physiological noise. Importantly, potential sources of 24 head motion parameters, as well as global, WM, and cerebrospinal fluid signals, were regressed to remove their effects [39].

### 2.8 Construction of Large‐Scale Functional Whole‐Brain Networks

The brain functional network for each patient was constructed first according to the Power atlas [40], which provides higher test–retest reliability for global and local network properties than the frequently used AAL atlas [41]. Of the original 246 regions of interest (ROIs) in the Power atlas, 227 were assigned to 10 brain networks in previous studies based on the original 13 networks [42]. The series for each ROI (spherical radius = 5 mm) were extracted by averaging the time courses of all the voxels within an ROI. Next, for each patient's condition, Pearson's correlation between the mean time series of each ROI was calculated. Then, a 246 * 246 symmetric correlation matrix for each patient was obtained, and the Fisher‐z transformation was applied to obtain the functional connectivity (FC) matrix.

Further, FC matrices were thresholded by connection density following previous graph‐based studies of brain connectivity [43]. Connection density equaled the number of existing edges divided by the maximal possible number of edges in a graph [44]. Our previous study investigated the cost range between 5% and 40% with intervals of 5%, as the graph becomes increasingly unstable and fragmented at lower costs and topology becomes increasingly random at higher costs [45].

### 2.9 Calculation of Topological Properties

Nodal properties, that is, nodal strength (NS) and nodal efficiency (NE), were calculated and analyzed as follows [46]:

NS is the sum of the edge weights of all the connections of a node:

$${\mathrm{NS}}_{i}=\sum_{j\in N}W_{\mathrm{ij}}$$

where *W*_*ij*_ is the edge weight between nodes *i* and *j*. It quantifies that a node is relevant to the graph.

NE is the inverse of the characteristic path length between a pair of nodes:

$${\mathrm{NE}}_{i}=\frac{1}{N-1}\sum_{j\neq i\in G}\frac{1}{L_{\mathrm{ij}}}$$

where *L*_*ij*_ is the weighted characteristic path length between nodes *i* and *j*.

### 2.10 Brain Functional Network Node Properties Analysis

The node topology attributes included NS and NE. (1) NS refers to the number of edges directly connected to a node and is a description of the statistical characteristics of interconnection between nodes; (2) NE is the average of the inverse of the average path length from the node to other nodes and is a description of the compactness of the node and its neighboring nodes.

### 2.11 Montreal Cognitive Assessment

All patients included in this study were assessed for cognitive function on the day of admission and before discharge using the MoCA scale. The MoCA scale evaluates overall cognitive function across 11 items in 8 cognitive domains: attention and concentration, executive function, memory, language, visual structural abilities, abstract thinking, calculation, and orientation. A total of 30 points were assigned to the MoCA scale, with ≥ 26 points considered normal, 18–26 points considered mild, 10–17 considered moderate, and less than 10 considered severe cognitive impairment. If the patient had ≤ 12 years of education (high school level), 1 point could be added to the score, but the total score could not exceed 30 points [15].

### 2.12 Statistical Analyses

Statistical evaluations were conducted utilizing IBM SPSS Statistics software, version 19.0 (IBM, Armonk, NY). Data distributions were graphically assessed using histograms, and their normality was evaluated using the Kolmogorov–Smirnov test. For variables that exhibited a normal distribution, comparisons between groups were made using two‐sample *t*‐tests. Non‐normally distributed variables were analyzed with the Mann–Whitney *U* test and the Kruskal–Wallis test. Categorical data were presented as counts (percentages) and compared via Chi‐Square tests or Fisher's exact tests when the expected frequency in any contingency table cell was < 5.

To address the nodal properties of the functional brain network, a two‐factor repeated measures ANOVA was conducted, considering subgroups for the presence of ischemia MMD and time factors (before and after anesthesia). If the main effect was significant, two‐tailed paired *t*‐tests for the time factor and two‐tailed two‐sample *t*‐tests for the group factor were used for within‐factor post hoc analyses. Statistical significance was set at *p* < 0.05, with false discovery rate correction applied. The correlation between MoCA scores and DMN was assessed using Spearman's rank correlation coefficient.

## 3 Results

### 3.1 General Patient Information

A total of 20 cases were included in this study: 10 patients with IMMD and 10 Control patients. There were no statistically significant differences in the demographic characteristics of patients in the two groups (*p* > 0.05) (Table 1).

**TABLE 1:** Comparative analysis of the demographic characteristics of patients in both groups.

	MMD group (*n* = 10)	Control group (*n* = 10)	*t*	*p*
Sex				
M	6 (60%)	5 (50%)		1.00
F	4 (40%)	5 (50%)		
Age (years)	43.53 ± 11.28	43.73 ± 13.33	−0.044	0.965
Height (cm)	165.53 ± 10.29	166.00 ± 10.02	−0.126	0.901
Weight (kg)	68.19 ± 9.81	69.23 ± 9.82	−0.292	0.772
ASA				
I	7 (70%)	9 (90%)		0.582
II	3 (30%)	1 (10%)		
Years of education(years)	13.07 ± 3.26	12.76 ± 3.21	0.263	0.794

*Note:* The data are shown as the number (percentage) unless otherwise indicated. Mean values are presented with the standard deviation.

Abbreviations: ASA, American Society of Anesthesiologists; F, female; M, male; MMD, Moyamoya disease.

### 3.2 Altered Node Topological Properties of Brain Networks in IMMD at the Awake State (Compared to the Control Group)

**FIGURE 1:**
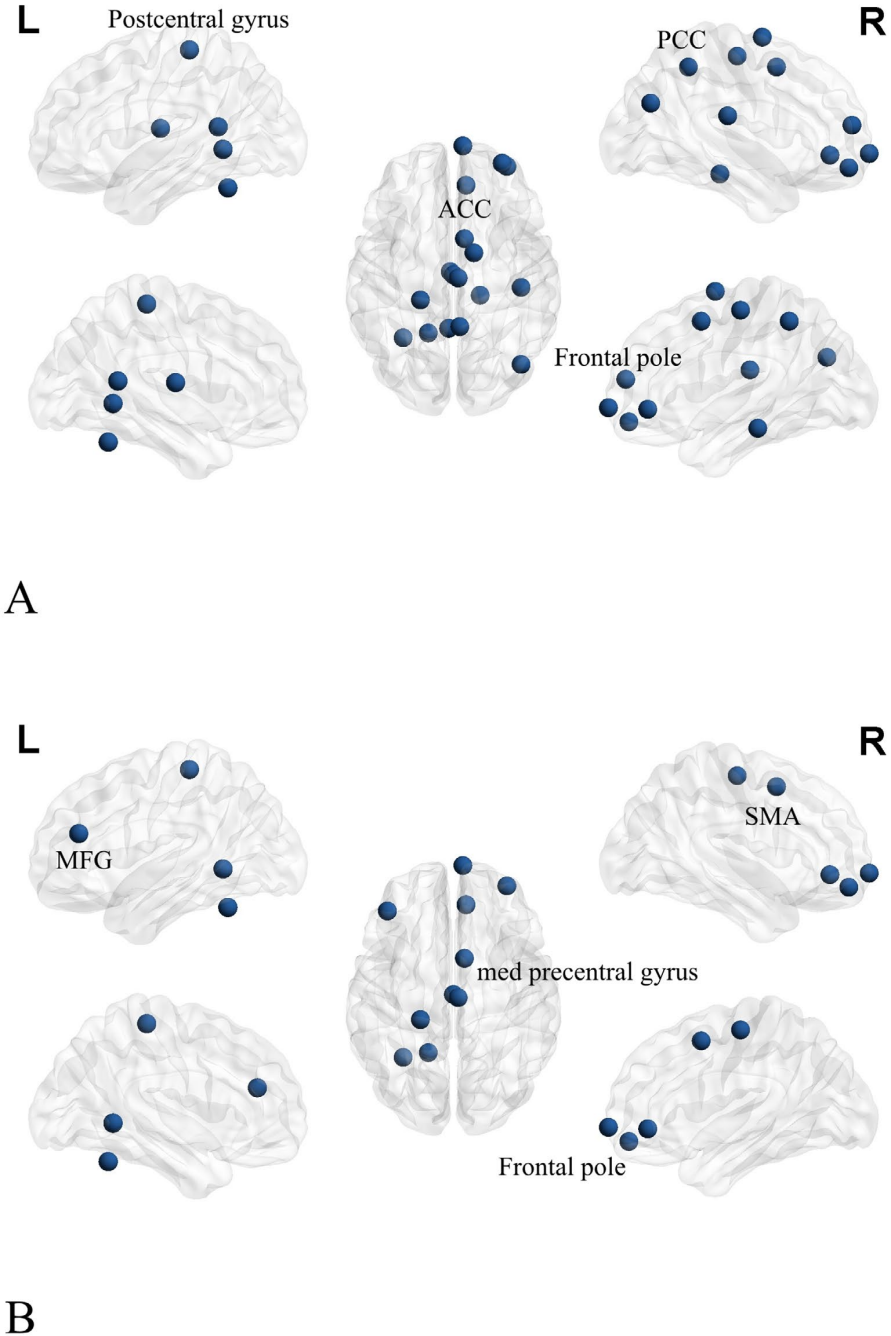
(A) NS analysis of IMMD in the awake state; (B) NE analysis of IMMD in the awake state (blue dots represent brain areas with a significant decrease in change). AC, anterior cingulate cortex; MFG, middle frontal gyrus; PCC, posterior cingulate cortex; SMA, supplementary motor area.

The investigation of altered local topological properties of brain networks in patients with IMMD using NS and NE revealed significant findings. Brain regions with significantly decreased NS in patients with IMMD compared to controls were mainly located in the DMN, sensory network (SEN), and FPN. The brain regions involved were the cingulate gyrus, postcentral gyrus, precentral gyrus, inferior frontal gyrus, motor auxiliary area, frontal pole, and posterior cingulate gyrus (Figure 1A). Brain networks in which the NE of IMMD was significantly decreased included the SAN, in addition to the DMN, SEN, and FPN. The specific brain regions were the precuneus, left middle frontal gyrus, and inferior frontal gyrus (Figure 1B).

### 3.3 Changes in Topological Properties of Nodes of IMMD Brain Networks Under Anesthesia

#### 3.3.1 IMMD Changes the Topological Properties of Brain Network Nodes Between Groups Under Anesthesia

**FIGURE 2:**
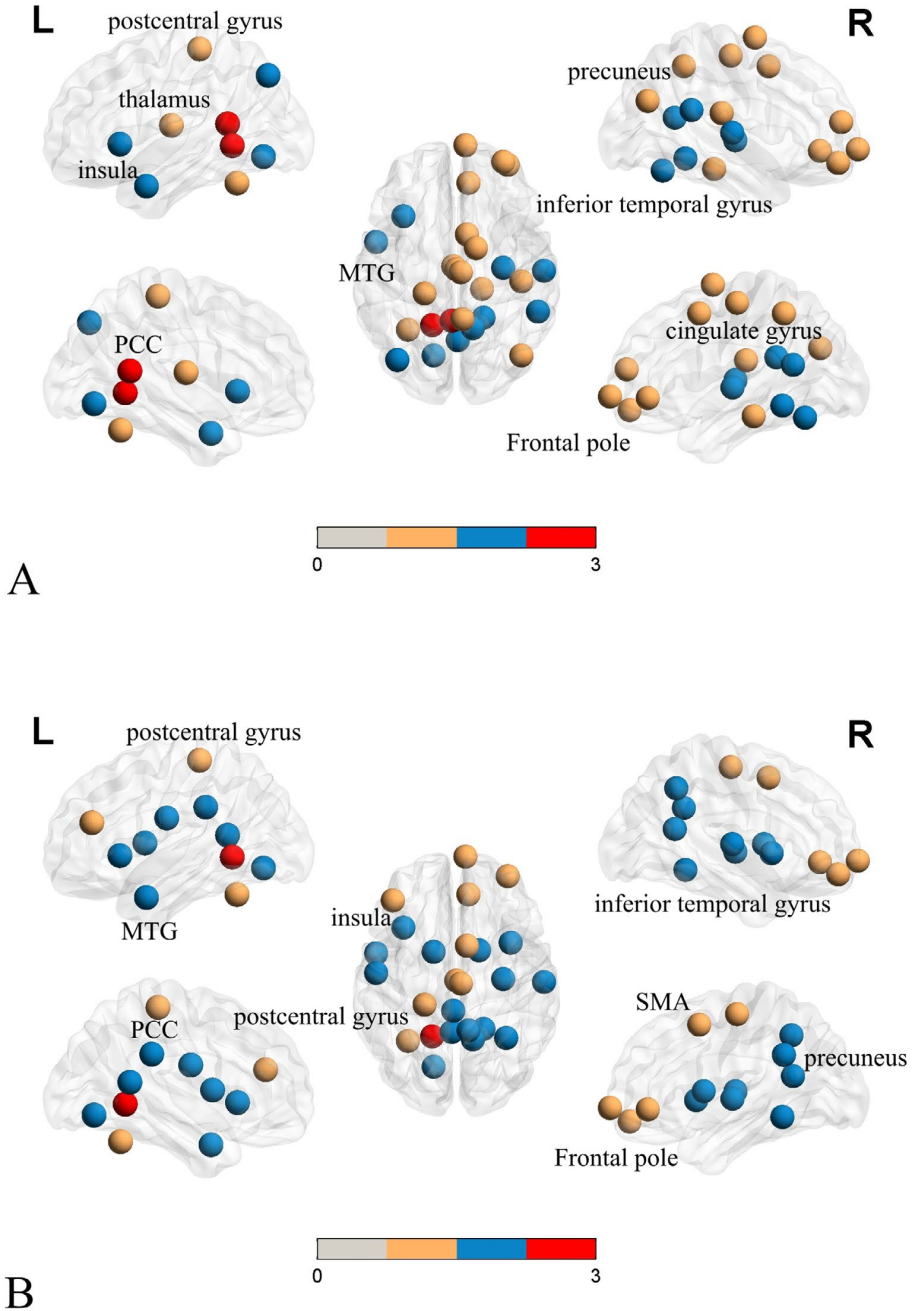
(A, B) Analyses of the topological properties of the brain complex nodes of IMMD under anesthesia; (A) Analysis under NS; (B) Analysis under NE (yellow dots represent difference before anesthesia, no difference after anesthesia; blue dots represent no difference before anesthesia, difference after anesthesia; red dots represent difference both before and after anesthesia). ACC, anterior cingulate cortex; MFG, middle frontal gyrus; MTG, middle temporal gyrus; PCC, posterior cingulate cortex.

To further elucidate the effects of IMMD and propofol on brain networks, we first explored the effects of disease on the between‐group differences. The altered local topological properties of brain networks in patients with IMMD were explored using NS and NE, and we consistently found that the significant decreases in the DMN, SEN, FPN, and cingulo‐opercular network occurring in patients with IMMD under anesthesia were likely related to the disease itself. IMMD may also lead to significant decreases in the cingulate gyrus, superior frontal gyrus, inferior temporal gyrus, and isthmic brain regions in this group of patients (Figure 2A).

#### 3.3.2 Alterations in the Topological Properties of Nodes of Intergroup Brain Networks Under Anesthesia by Propofol

We further analyzed the changes due to propofol on the NS and NE of both groups under anesthesia. The results revealed that the significant decreases in the brain regions of the DMN, SEN, FPN, and SAN in patients in the IMMD group might be related to the effects of propofol. The specific decreased brain regions included the temporal pole, posterior cingulate gyrus, middle temporal gyrus, angular gyrus, and inferior temporal gyrus (Figure 2B).

### 3.4 Changes in Topological Properties of Brain Network Nodes Before and After Anesthesia in the IMMD Group

**FIGURE 3:**
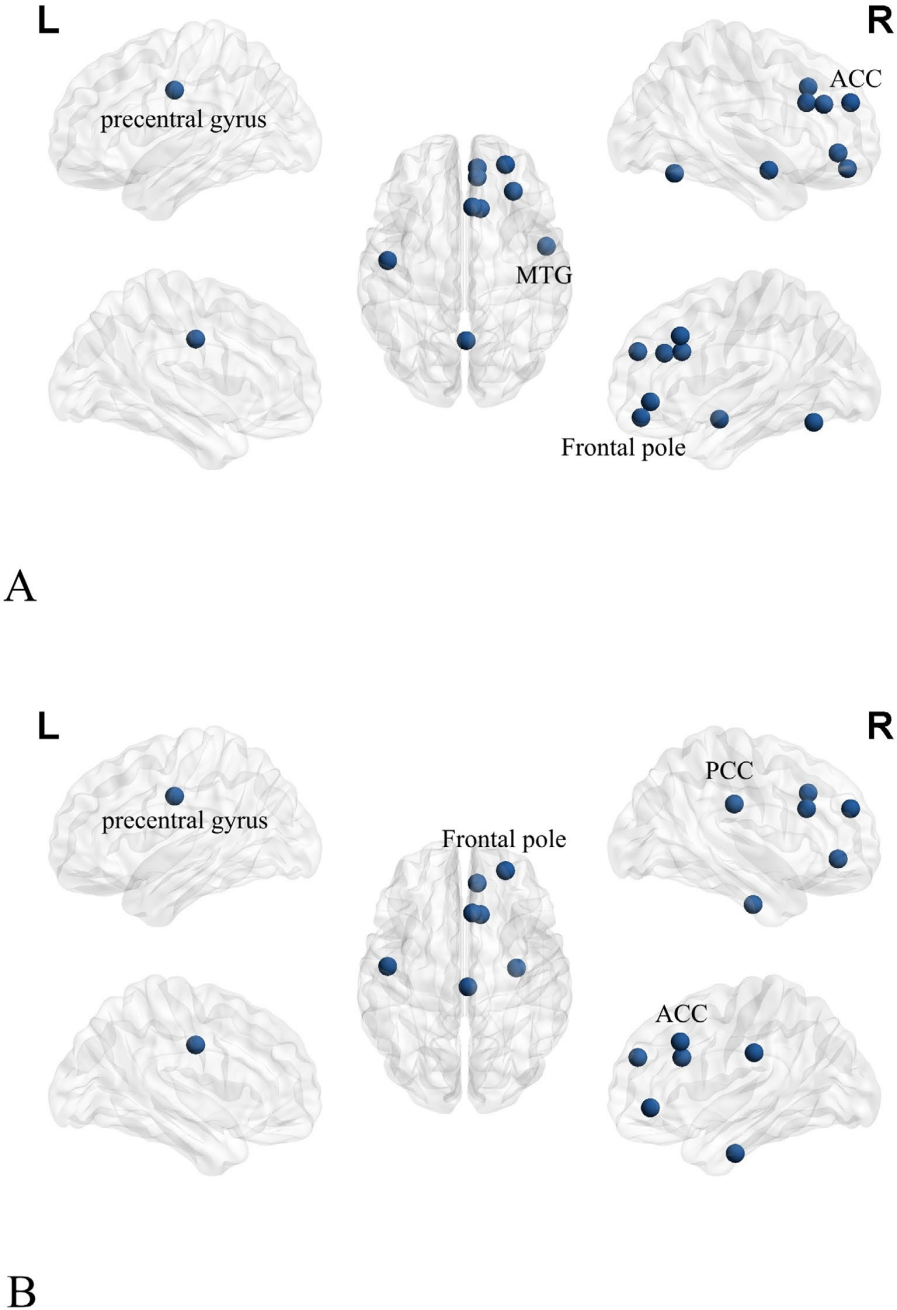
(A, B) Analyses of topological properties of brain complex nodes in IMMD before and after anesthesia; (A) Analysis under NS; (B) Analysis under NE (blue dots represent brain areas with a significant decrease in changes). MFG, middle frontal gyrus; MTG, middle temporal gyrus; PCC, posterior cingulate cortex; SMA, supplementary motor area.

After exploring using NS and NE, we consistently found that patients with IMMD experienced significant reductions in the medial frontal gyrus in the DMN, as well as the left middle frontal gyrus, middle temporal gyrus, anterior cingulate cortex, central frontal region, and precuneus occurring in the SAN before and after propofol anesthesia (Figure 3A,B).

### 3.5 Comparing the MoCA Scale Between the Two Patient Groups at Two Time Points

The MoCA scale scores of patients in the Control group were higher than those in the IMMD group, and the difference was statistically significant (*p* < 0.05), as shown in Table 2.

**TABLE 2:** Comparison of MoCA scores at the time of admission and discharge between the two groups of patients.

	MMD group	Control group	*t*	*p*
Hospitalization	18.14 ± 1.96	26.53 ± 1.70	−12.775	0.000
Discharge from hospital	19.57 ± 2.85	26.12 ± 1.54	−7.725	0.000

### 3.6 Correlation Between DMN and MoCA Scores at Admission and Discharge

**FIGURE 4:**
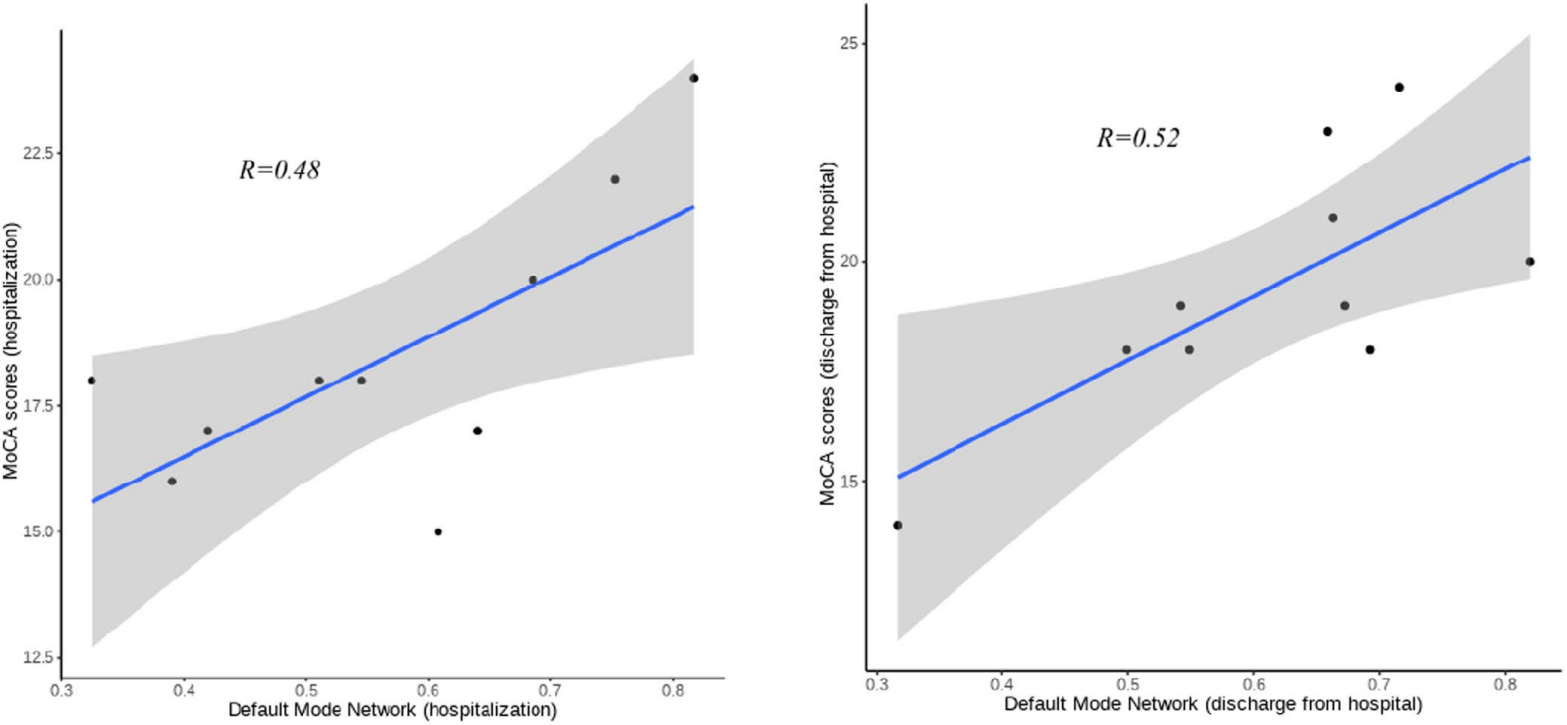
Correlation between DMN and MoCA scores at admission and discharge.

The DMN at the time of patient admission and discharge had a moderately strong correlation with MoCA scores, with correlation coefficients of 0.48 and 0.52, respectively (Figure 4).

## 4 Discussion

Changes in IMMD brain networks may be affected by propofol and remifentanil under general anesthesia. In this study, we applied rs‐fMRI to analyze brain functional network pre‐ and post‐anesthesia in patients with IMMD to explore the effects of these general anesthetic agents in these patients. In the awake state, patients with MMD have abnormal functional brain connectivity and functional network topological properties [47]. Rs‐fMRI and arterial spin labeling show that patients with IMMD have fewer functional connections, lower frontal and temporal centrality, and increased cerebellar compensation [4]. Executive functions, including semantic inhibition, executive processing, working memory, and sustained attention, are significantly impaired in adults with IMMD, mainly due to stenosis, which reduces brain network connectivity, damages brain network structure, and affects related brain regions, ultimately leading to cognitive decline [48]. Decreased frontal perfusion may be associated with working memory and semantic inhibition deficits in patients with IMMD [49]. Our comparison of the two groups identified the cingulate gyrus, posterior central gyrus, precentral gyrus, inferior frontal gyrus, motor accessory area, frontal pole, posterior cingulate cortex, precuneus, left middle frontal gyrus, and inferior frontal gyrus as regions with significantly decreased brain functional connectivity in the IMMD group before anesthesia. Most of these regions are associated with cognition and consciousness [43], consistent with previous findings of brain region changes in patients with IMMD [27, 50, 51].

In a comparative study with healthy controls, Liu et al. [52] found that patients with cerebral small vessel disease (CSVD), both with and without cognitive impairment, had reduced functional connectivity within the DMN, and functional connectivity between the DMN and the dorsal attentional network (DAN) and the FPN also showed a reduced negative correlation. Interaction between these networks is critical for attention and executive function, with the DMN playing a central role [53]. Our results revealed significant reductions in DMN, sensorimotor network (SMN), FPN, and SAN functioning among patients with IMMD, which modulate awareness, cognition, and executive performance. These findings were consistent with the clinical presentation of patients with IMMD, particularly the DMN, which had the highest correlation with cognitive effects. The DMN at the time of patient admission and discharge had a moderately strong correlation with MoCA scores (*r* = 0.48 and 0.52).

Previous studies have linked structural damage to brain networks in CSVD and global disease burden scores, as well as cognitive deficits. These deficits manifest as global cognitive impairment, executive dysfunction, and memory issues, and correlate with the volume of WM hypoplasia (WMH), local structural network efficiency, and information processing speed [54]. Further brain network studies using rs‐fMRI have confirmed that the cognitive deficits seen in patients with CSVD are associated with impairments in large‐scale functional networks such as the DMN, FPN, and DAN [55]. CSVD specifically affects the DMN, DAN, and FPN. Our comparison of patients with IMMD before and after propofol combined with remifentanil anesthesia revealed significant reductions in the medial frontal gyrus in the DMN, the left middle frontal gyrus, the middle temporal gyrus, the anterior cingulate cortex, the central frontal region, and the precuneus in the SAN. This suggests that the DMN and the SAN remain the primary brain networks affected by anesthesia in IMMD and that both networks play a crucial role in cognitive functions. The SAN, anchored in the anterior insula cortex, has been hypothesized to modulate the activity of the DMN during stimulus‐driven cognitive processes [56], which may have an impact on cognitive function in patients with IMMD after anesthesia.

Changes in brain function after anesthesia in patients with IMMD have rarely been studied. Propofol, the general anesthetic used in this study, is a sedative‐hypnotic drug widely used in neurosurgery for its ability to modulate the excitatory amino acid transmitter system and protect brain cells from oxidative stress, thereby exerting neuroprotection [57, 58]. However, its sedative‐hypnotic effects have some effects on functional brain networks, as indicated by decreased thalamic, frontoparietal, and DMN connectivity during propofol anesthesia [23, 59, 60]. As the dosage of propofol was increased, a significant change in the brain network's connectivity was observed. This change was attributed to the propofol‐induced enhancement of the GABA receptor inhibitory effect, which suppresses neuronal activity and shifts consciousness from wakefulness to unconsciousness. The observed effects of the drug manifested as a decline in the brain's capacity for integrated function, particularly evident through a reduction in connectivity within critical areas such as the thalamocortical network. Concurrently, propofol induced a decrease in cerebral blood flow and metabolic rate, further impacting functional connectivity between disparate brain regions. These alterations collectively elucidate the impact of propofol on the dynamic reorganization of brain networks, a process that is indispensable for sustaining consciousness and perception [26]. Decreased functional connectivity has been observed in the frontal cortex and anterior temporal cortex during propofol‐induced loss of consciousness [29] as have decreased functional connectivity in the posterior cingulate cortex and anterior temporal cortex. The posterior cingulate cortex and precuneus are significantly inhibited by propofol, resulting in deficits in cognitive function and information integration, leading to loss of consciousness [24].

The general anesthetic analgesic used in this study was a targeted infusion of remifentanil. Previously identified brain regions most affected by pain were the bilateral insular cortex and the anterior cingulate cortex, whereas one of the main brain regions modulated by remifentanil was the right insular cortex [28], which was largely unrelated to the brain regions in this study. Remifentanil is an ultrashort‐acting μ‐opioid receptor agonist that has no significant effect on neurovascular activity, particularly in brain regions outside the pain matrix [35]. Therefore, this study excluded the effects of remifentanil on brain networks and regions. We found that patients with IMMD under propofol combined with remifentanil anesthesia showed decreases in the DMN, SEN, FPN, and SAN, consistent with the brain networks altered in the awake state among patients in the IMMD group, which was not found in any previous studies. However, whether this method of general anesthesia affects the cognitive function of patients with IMMD and the degree of this effect requires confirmation with cognitive and executive function assessment and longer follow‐up in future studies.

It remains unclear whether the brain network changes caused by the IMMD disease itself exacerbate the alterations in brain network observed under anesthesia. A two‐way ANOVA designed to exclude the influence of propofol revealed that brain networks showing alterations after anesthesia due to the disease itself mainly included the DMN, FPN, SMN, and CON. These regions are generally related to cognitive and executive functions. In this prospective study, we described the changes in brain networks in patients with IMMD at rest as well as after anesthesia with propofol combined with remifentanil by rs‐fMRI, and we investigated the global differences of whole‐brain functional networks between the IMMD and control groups using graph‐theoretic analysis. The results showed that the brain networks affected by the disease in patients with IMMD itself were essentially the same as those affected after anesthesia with propofol combined with remifentanil. These results provide valuable insights for exploring the changes in brain networks of cognitive dysfunction in patients with IMMD and the effects of general anesthetic agents (propofol combined with remifentanil) on functional brain networks, suggesting a more appropriate anesthetic regimen for patients with IMMD.

### 4.1 Limitations

This study has some limitations. First, the sample size is relatively small, and data bias due to inter‐individual variation may influence the results of statistical analyses, which may be counteracted by an increase in the number of collections. Second, cognitive function was assessed solely at the time of patients' admission and discharge, and longer follow‐up data were unavailable. Changes in brain networks and the recovery of cognitive function may be a dynamic process that necessitates a more extended observation period. Third, although there were no differences in MoCA scores in the MMD group between the time of admission and discharge, there was a decrease in DMN connectivity. This may be due to a mismatch between the reduced cerebral functional connectivity and the increased cognitive function scores as a result of increased perfusion on the affected side after STA‐MCA bypass surgery.

In conclusion, according to the graphical analysis, we found that the whole‐brain functional networks and related brain regions with reduced brain functional connectivity were similar in patients with IMMD before and after anesthesia by the effects of systemic intravenous drugs. It was related to affecting consciousness, cognition, and executive ability, such as the DMN, the FPN, and the SEN. Significant decreases in brain connectivity were observed in regions such as the medial frontal gyrus, the left median frontal gyrus, the middle temporal gyrus, the anterior cingulate cortex, the central frontal region, and the precuneus. This suggests that intravenous general anesthetics (propofol combined with remifentanil) impact the whole‐brain functional network in IMMD and may help anesthesiologists develop appropriate anesthesia protocols for this patient population. We hope to increase the sample size, implement multicenter study designs, and incorporate additional covariate controls in future research to validate these findings and reduce potential bias.

## Author Contributions

Clinical data were collected by S.W. and X.Q. The data were analyzed and interpreted by Z.L. and X.L. X.C. and X.Q. drafted the manuscript, while L.Y. and X.G. provided critical revisions to ensure the accuracy of the knowledge content. Z.L. and H.Z. performed statistical analysis of the data. Supervision and validation of the manuscript were performed by R.D., R.W., and X.G. All authors have reviewed and approved the final manuscript.

## Ethics Statement

Ethical approval was granted by the Peking University International Hospital's institutional review committee, and all patients signed the consent form (Approval No. 2022‐KY‐0016‐01).

## Consent

All participants provided written informed consent for this study.

## Conflicts of Interest

The authors declare no conflicts of interest.

## Data Availability

The data that support the findings of this study are available from the corresponding author upon reasonable request.
